# A Novel Fuzzy Controller for Visible-Light Camera Using RBF-ANN: Enhanced Positioning and Autofocusing

**DOI:** 10.3390/s22228657

**Published:** 2022-11-09

**Authors:** Junpeng Zhou, Letang Xue, Yan Li, Lihua Cao, Changqing Chen

**Affiliations:** Changchun Institute of Optics, Fine Mechanics and Physics, Chinese Academy of Sciences, Changchun 130033, China

**Keywords:** camera control system, RBF ANN fuzzy control algorithm, continuous zoom, focal length fitting, autofocusing

## Abstract

To obtain high-precision for focal length fitting and improve the visible-light camera autofocusing speed, simultaneously, the backlash caused by gear gaps is eliminated. We propose an improved RBF (Radical Basis Function) adaptive neural network (ANN) FUZZY PID (Proportional Integral Derivative) position closed-loop control algorithm to achieve the precise positioning of zoom and focus lens groups. Thus, the Levenberg–Marquardt iterative algorithm is used to fit the focal length, and the improved area search algorithm is applied to achieve autofocusing and eliminate backlash. In this paper, we initially adopt an improved RBF ANN fuzzy PID control algorithm in the position closed-loop in the visible-light camera position and velocity double closed-loop control system. Second, a similar triangle method is used to calibrate the focal length of the visible-light camera system, and the Levenberg–Marquardt iterative algorithm is used to fit the relation of the zoom potentiometer code values and the focal length to achieve the zoom position closed-loop control. Finally, the improved area search algorithm is used to achieve fast autofocusing and acquire clear images. The experimental results show that the ITAE (integrated time and absolute error) performance index of the improved RBF ANN fuzzy PID control algorithm is improved by more than two orders of magnitude as compared with the traditional fuzzy PID control algorithm, and the settling time is 6.4 s faster than that of the traditional fuzzy PID control. Then, the Levenberg–Marquardt iterative algorithm has a fast convergence speed, and the fitting precision is high. The quintic polynomial fitting results are basically consistent with the sixth-degree polynomial. The fitting accuracy is much better than that of the quadratic polynomial and exponential. Autofocusing requires less than 2 s and is improved by more than double that of the traditional method. The improved area search algorithm can quickly obtain clear images and solve the backlash problem.

## 1. Introduction

Visible-light cameras have been widely used in photoelectric tracking turntables [1]. In the common closed-loop control system of visible-light camera positions, a simple PID control algorithm is used to achieve mirror group precision positioning. To improve control precision and robustness performance, a large number of advanced control methods have been provided in the field of automatic control. The control strategies mainly include ILC (iterative learning control) and MPC (model predictive control) [2], sliding mode control [3], active disturbance rejection method [4,5], H∞ [6], nonlinear disturbance observer [7,8], etc. However, the most widely implemented industrial controllers are currently based on PID algorithms to expand, such as LQR (linear quadratic regulator)-PID controller applied in intelligent vehicles [9] and a new adaptive sliding mode control method based on the RBF neural networks introduced in a robotic excavator [10]. Meanwhile, the neural network fuzzy PID control improved dynamic performance in a brushless DC motor control system. Finally, considering the high practicability and reliability in the DSP (Digital Signal Processing) servo control system [11,12] to improve the dynamic performance of the PID control algorithm, a fuzzy PID control algorithm is proposed. The proportional coefficient $k_{p}$, integral coefficient $k_{i}$, and differential coefficient $k_{d}$ are obtained according to engineering experience; thus, it requires considerable time to obtain [13]. After the accurate positioning of the camera lens group, focus and zoom control can be completed. It is necessary to adjust the focal length and focal plane to obtain a clear image when tracking a long-distance target. Meanwhile, the passive ranging function is achieved by calibrating the camera focal length to obtain three-dimensional information about the target [14,15]. The calibration camera focal length mainly includes the parallel light focusing method, Gaussian formula method, secondary imaging method, lateral magnification method, autocollimation method, and node method [16,17,18]. However, three points influence the accuracy of measuring the focal length. First, the lens focal depth results in the image plane position uncertainty. Second, the existence of the lens spherical aberration causes noncoincidence in the visual image plane and Gaussian image plane. Finally, the thickness of the lens is not real to zero. Moreover, there is no method to avoid or reduce it directly through experiments, and it only depends on theoretical analysis for correction [19,20]. Therefore, we used the Gaussian formula method in this project to calibrate the focal length. When tracking a long-distance target at a specified focal length, the target is far away from the field of view, and the image becomes blurred. Autofocusing technology is used to quickly focus and recapture the target. In principle, autofocusing technology can be divided into passive focusing and active focusing. The main idea is to adjust the focal plane position to achieve clear imaging according to the optical Gaussian formula [21,22,23,24]. Some studies have suggested that deep predictive zoom tracking algorithms achieve autofocusing. Although it has a higher prediction accuracy and can generate accurate trace curves, it greatly depends on computers and needs more parameters. This algorithm cannot be implemented in low-cost DSP and is not conducive to project realization [25,26]. The method consists of a rough focusing step and a fine focusing step that are applied to autofocusing to obtain a high-definition picture; however, autofocusing speed is not yet considered [27,28]. Although the mountain-climb method can quickly and accurately obtain clear images, the image background is not complete. For this problem, we propose applying a four-layer fuzzy RBF adaptive neural network to calculate the PID parameters in place of the traditional fuzzy PID control algorithm $k_{p}$, $k_{i}$ and $k_{d}$ terms and combine them with the fuzzy PID control to improve the dynamic performance. Moreover, the Levenberg–Marquardt algorithm [29] is introduced in this project, fitting the relation between the zoom code values and focal length, and we improved the area search method to achieve fast autofocusing. It can also solve the poor focusing problem resulting from the gear gap.

## 2. Visible-Light Camera System Hardware Structure

**Figure 1:**
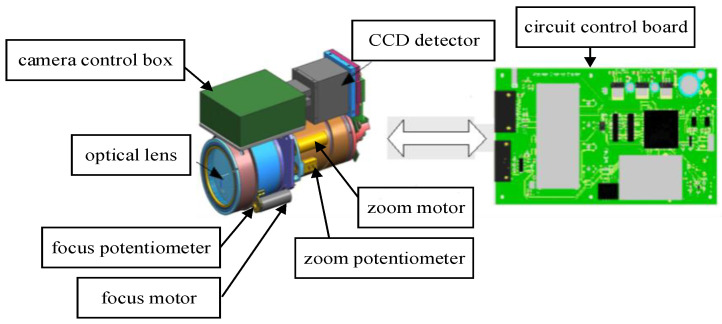
Visible-light camera hardware structure.

The F25 mm–500 mm visible-light camera zoom system hardware consists of the zoom lens, the optical lens, a circuit control board, a CCD (charge-coupled device) detector, a focusing potentiometer, a zoom potentiometer, a focusing miniature DC motor and a zoom miniature DC motor. The basic structure of the zoom system is shown in Figure 1.

The zoom motor drives the zoom lens group through the gear to transform the field of view. The focusing motor drives the focusing lens group to show a clear image. The zoom and focusing potentiometer are the position feedback elements that output analog voltage signals after filtering and are sent to the DSP 28335 AD module to obtain potentiometer position feedback code values. The position feedback differential operation obtains the velocity feedback signal. The fitting feedback focal length is derived from the potentiometer code values and the measuring focal length.

## 3. Visible-Light Camera Control Algorithm

### 3.1. Traditional Fuzzy PID Control Algorithm

**Figure 2:**
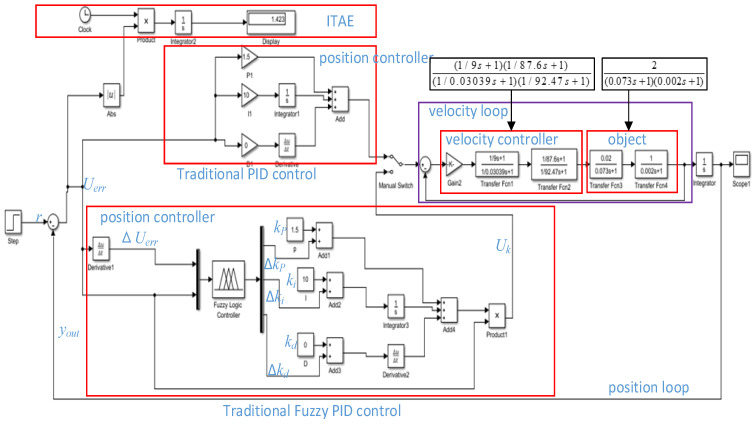
Position and velocity double closed-loop control diagram.

The camera servo system adopts position and velocity double closed-loop control systems, the position controller uses a fuzzy PID algorithm, and the velocity loop uses a second-order controller. The control structure is shown in Figure 2.

In Figure 2, the object model of the camera control system is $\frac{2}{\left(0.073s+1)\times (0.002s+1\right)}$ , which is obtained from the velocity open-loop step response [1]. The velocity controller is $\frac{\left(1/9s+1)\;(1/87.6s+1\right)}{\left(1/0.03039s+1)\;(1/92.47s+1\right)}$ [1], the performance index is $ITAE=\int t\times \left|e(t)\right|d_{t}$ [11], $U_{err}$ is the position error signal, $\Delta U_{err}$ is the position error differential signal, r is the position input signal, and $y_{out}$ is the position output signal, which is collected through the precision multiturn potentiometer. The potentiometer outputs the position voltage, which is fed into the DSP28335 AD module as a feedback signal after filtering, and the position signal differential operation obtains a velocity feedback signal. In this paper, the filter are double inertial elements, and its time constant is selected by the bandwidth of the potentiometer outputs voltage. The feedback signal $y_{out}$ and the given zoom or focusing instruction r undergo the differential operation. The outcome is used as the input of the position controller to obtain the output $U_{k}$ of the position controller, which will serve as the input of the velocity loop. The input of the velocity loop and the feedback velocity undergo the differential operation to yield the input of the velocity controller. Finally, we obtain the position output by performing the integral operation that constitutes the camera servo control system. However, the DSP 28335 AD module collecting feedback potentiometer values is a 16-bit digital signal, which cannot be used directly for zoom feedback control. It is necessary to convert the code values of the potentiometer feedback into focal length values to obtain the position closed-loop control.

**Figure 3:**
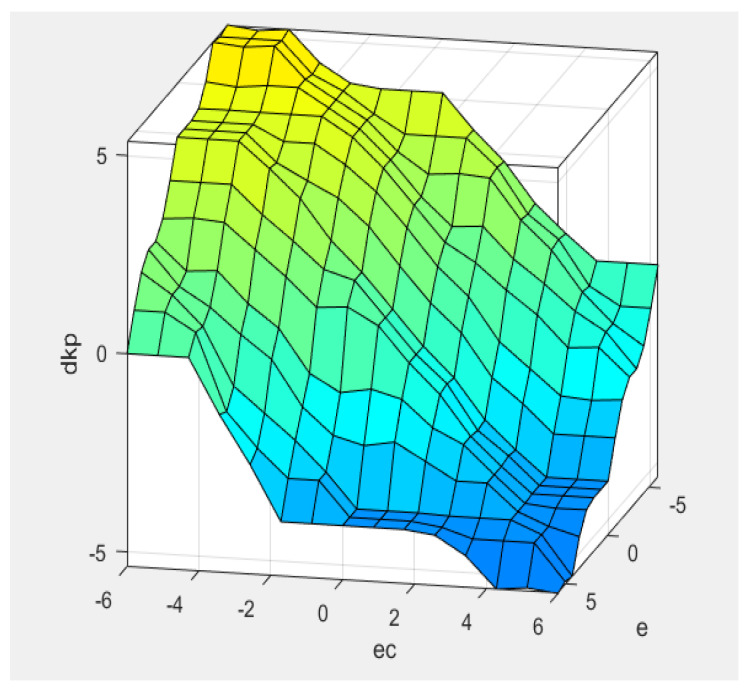
$\Delta k_{p}$ output surface.

**Figure 4:**
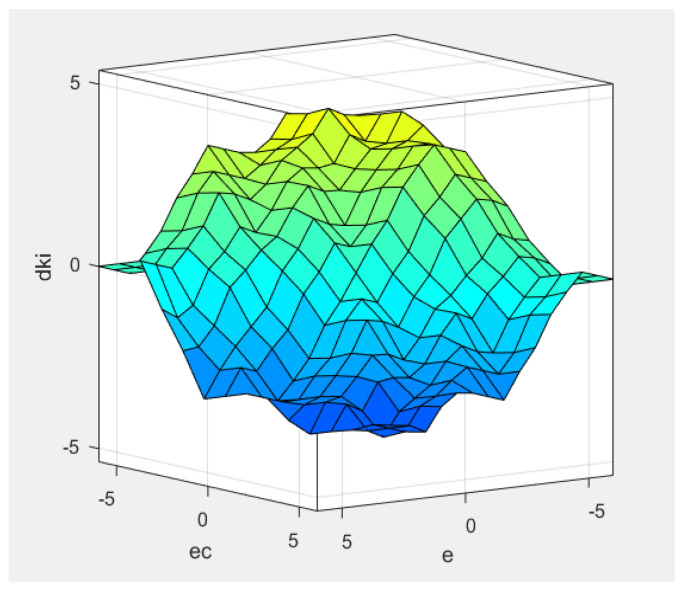
$\Delta k_{i}$ output surface.

**Figure 5:**
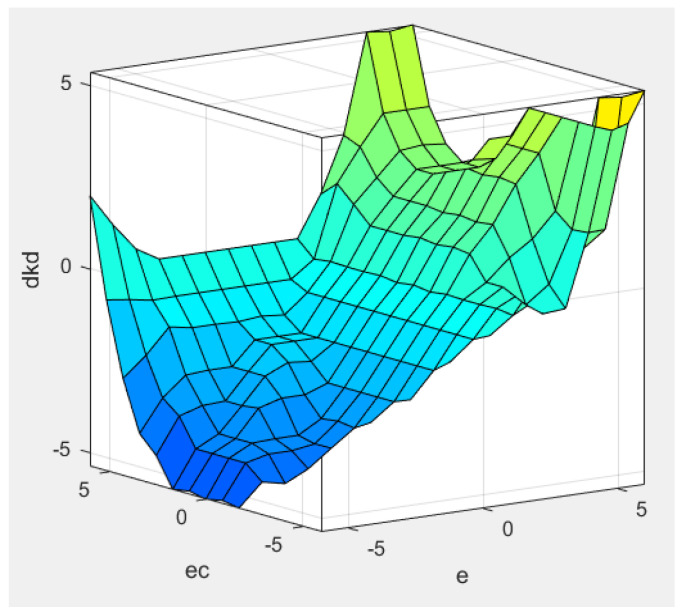
$\Delta k_{d}$ output surface.

In Figure 2, we set the position error and position error variation as the fuzzy input of the fuzzy logic controller in the position loop and set the proportional variation $\Delta k_{p}$, integral variation $\Delta k_{i}$ and differential variation $\Delta k_{d}$ as the fuzzy output. The context range of all input and output are from −6 to 6. The fuzzy subset of the output $U_{err}$ and $\Delta U_{err}$ are seven elements [NB, NM, NS, Z, PS, PM, PB], the fuzzy membership function adopts a trigonometric function, the fuzzy rules table uses 49 traditional fuzzy rules, and defuzzification is the centroid algorithm. Through the fuzzy logic controller simulation in Figure 2, the output surface of the fuzzy control quantities $\Delta k_{p}$, $\Delta k_{i}$, and $\Delta k_{d}$ can be obtained as shown in Figure 3, Figure 4, and Figure 5, respectively [30].

A formula is as follows from Figure 2:

$$U_{k}(t)=(k_{p}+\Delta k_{p})U_{err}(t)+\int (k_{i}+\Delta k_{i})U_{err}(t)dt+(k_{d}+\Delta k_{d})dU_{err}(t)/dt$$

where the proportional coefficient $k_{p}$, integral coefficient $k_{i}$, and differential coefficient $k_{d}$ are obtained through engineering experience.

### 3.2. Improved RBF ANN Fuzzy PID Control Algorithm

**Figure 6:**
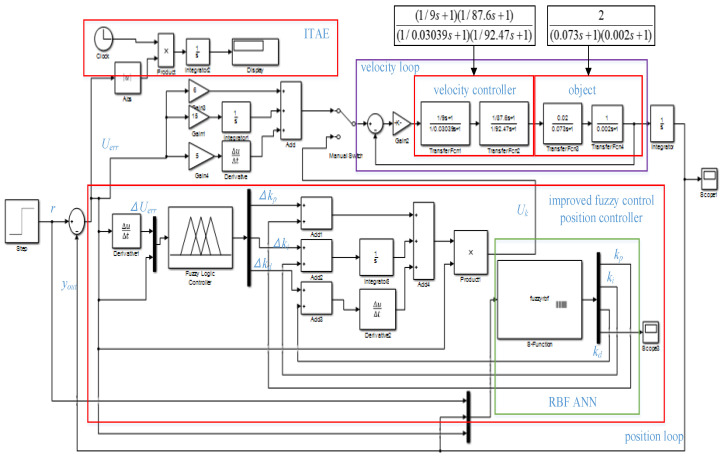
RBF ANN fuzzy PID control system.

The traditional fuzzy PID control algorithm is better than the ordinary PID control [30]. However, adjusting $k_{p}$, $k_{i}$, and $k_{d}$ requires considerable time. Therefore, we propose applying a four-layer fuzzy RBF ANN to calculate the PID parameters in place of the traditional fuzzy PID control algorithm $k_{p}$, $k_{i}$, and $k_{d}$ terms and combine them with the fuzzy PID control. We utilized S-Function to compile an RBF ANN program in Matlab. Finally, a new position regulator is constructed. A novel control system is shown in Figure 6.

The design of the fuzzy PID controller in Figure 6 is consistent with that of Section 3.1, so it will not be described again. The input vectors of the four-layer fuzzy RBF ANN controller are taken as two vectors. Every input vector corresponds to 5 fuzzy sets for fuzzification, and the outputs are the three vectors of the proportional coefficient $k_{p}$, integral coefficient $k_{i}$ and differential coefficient $k_{d}$. The structure of the four-layer fuzzy RBF neural network is 2-5-5-3. The four layers of the fuzzy RBF neural network are as follows:

The input layer is $f_{1}(i)=[r,y_{out}]$, where i=2, r is the input, and $y_{out}$ is the output.

The fuzzified layer is $f_{2}(i,j)=\exp(-\frac{{\left(f_{1}(i)-C_{ij}\right)}^{2}}{b_{j}{}^{2}})$, where i=2, j=5, $C_{ij}=0.3\times ones(2,5)$ is the mean value of membership function, an $b_{j}$ is the standard deviation of the membership function fuzzy sets and $b_{j}=ones(5,1)$. The $f_{2}$ matrix is $f_{2}=\left[\begin{array}{ccc} f_{2}(1,1) & \cdots & f_{2}(1,5)\\ f_{2}(2,1) & \cdots & f_{2}(2,5) \end{array}\right]$.

The fuzzy inference layer is $f_{3}(j)=\prod_{k=1}^{2}f_{2}(i,j)$. where $f_{3}(1)=f_{2}(1,1)\times f_{2}(2,1)$, $f_{3}(2)=f_{2}(1,2)\times f_{2}(2,1)$…, $f_{3}(25)=f_{2}(1,5)\times f_{2}(2,5)$.

The output layer is $f_{4}[kp,ki,kd]=\omega \times f_{3}=\sum_{j=1}^{25}\omega (i,j)\times f_{3}(j)$, where the network weight ω iteration formula is as follows [13]:

(1) $$\omega_{j}(k)=\omega_{j}(k-1)+\Delta \omega_{j}(k)+\alpha (\omega_{j}(k-1)-\omega_{j}(k-2))$$

(2) $$\Delta \omega (i,j)=\eta \times U_{err}(k)\times dyu(k)\times XC(j)\times f_{3}(i)$$

(3) $$dyu(k)=sign(y_{out}-y_{out}(k-1))/(du(k)-du(k-1)+0.0001)$$

(4) $$du(k)=f_{4}\times XC^{T}$$

(5) $$XC(k)={\left[U_{err}(k)-U_{err}(k-1),U_{err}(k),U_{err}(k)-2U_{err}(k-1)+U_{err}(k-2)\right]}^{T}$$

(6) u(k)=u(k−1)+du(k)

where the initial value is $\omega_{0}=rands(3,25)$, j=3, i=25, k is the iteration, the web-based learning parameters are η=0.2 and α=0.02, u(k) is control quantity, $U_{err}(k)=r(k)-y_{out}(k)$ and $f_{3}(i)$ is the calculated vector from the fuzzy inference layer.

By improving the fuzzy PID control algorithm of the RBF neural network, we can see that there is no need to adjust the parameters $k_{p}$, $k_{i}$, and $k_{d}$ according to engineering experience. If the RBF adaptive neural network and fuzzy PID neural network are set, the PID parameters can be adjusted online in the position loop. The design process of the position regulator is simplified, and the design time is reduced.

### 3.3. The Focal Length Fitting Algorithm

#### 3.3.1. Camera Focal Length Calibration

We place the parallel light pipe in front of the camera. The parallel light pipe focal length is 550 mm, and the eyepiece has a fixed 4 mm scale line at the end of the parallel light pipe. The camera characteristics in this system are shown in Table 1.

**Figure 7:**
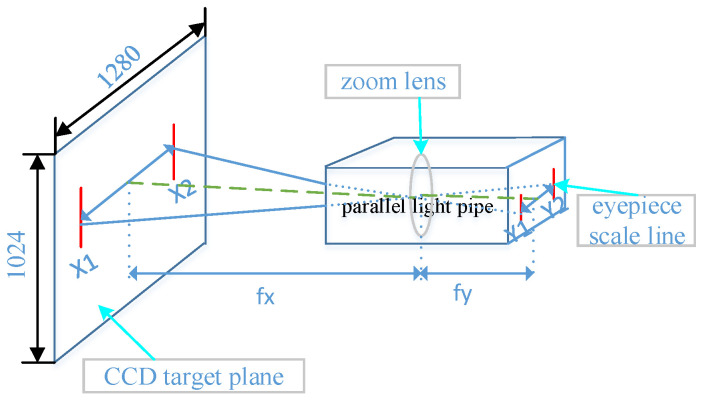
Measuring focal length diagram.

**Figure 8:**
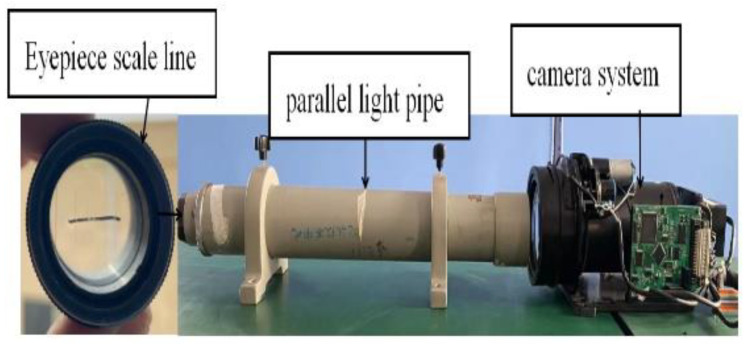
Measuring focal length physical figure.

The lines on the eyepiece are displayed on the CCD target plane through the zoom lens group. Finally, the focal length of the visible-light camera system can be calculated through a similar triangle. The focal length calibration diagram and physical figure are shown in Figure 7 and Figure 8, respectively.

In Figure 7, the target scale lines imaging on the target plane of the CCD camera are X1 and X2. Y1 and Y2 are the eyepiece scale lines. $f_{y}$ is the focal length of the parallel light pipe. Therefore, the focal length of the visible-light zoom lens group can be calculated through a similar triangle. Finally, from Figure 7, we can obtain Formula (7).

(7) $$fx=\frac{(X2-X1)\times 4\times fy}{Y2-Y1}$$

The focal length of the parallel optical pipe and the eyepiece scale lines have been accurately calibrated. It can be known from Formula (7) that the focal length measurement error mainly comes from X1 and X2. The eyepiece scale lines are clearly imaged on the target plane. The pixel size is μm level, and when compared with the focal length mm level, the measurement error can be ignored. The distance between X1 and X2 can be calculated accurately by imaging the target plane, Y2−Y1=4 mm, $f_{y}=550\;\mathrm{mm}$, so we can calculate the camera focal length $f_{x}$.

#### 3.3.2. Relation of the Focal Length and Zoom Potentiometer Code Values

**Figure 9:**
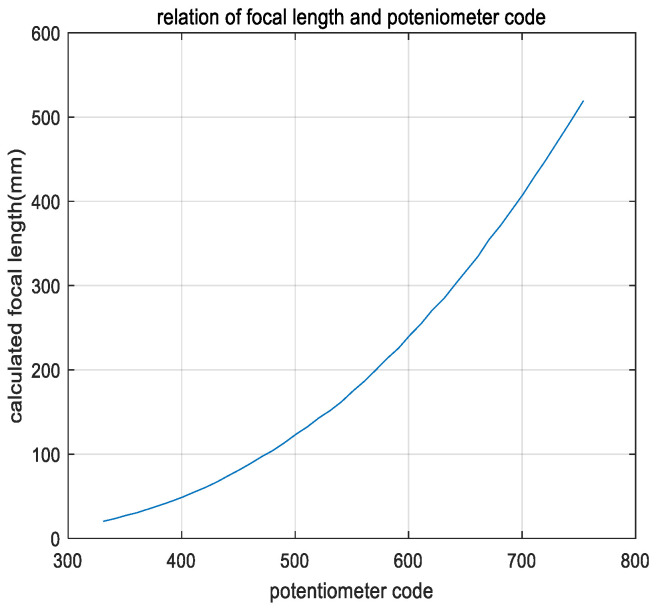
Relation of the zoom potentiometer code values and focal length.

The DSP28335 AD module collects the position code values of the feedback potentiometer for continuous zoom position closed-loop control, which is not the focal length value. Therefore, we need to obtain the relation between the focal length and potentiometer code values; however, the relation between the focal length and potentiometer code values is nonlinear. Therefore, the potentiometer code values are used to calculate the feedback focal length by a fitting algorithm in the DSP to achieve the zoom position closed-loop control. In this project, the range of the zoom potentiometer feedback code values collected by the DSP28335 AD module is from 331 to 754. To ensure a high fitting accuracy, 10 interval points are used to calculate a focal length value through Formula (7). Finally, the number of calculations of the focal length values was 43. The relation between the actual measured focal length and code values is shown in Figure 9.

From Figure 9, we can see that the minimum focal length is 20.24 mm, and the corresponding AD code value is 331. The maximum focal length is 519.20 mm, and the corresponding AD code value is 754. It fully covers the focal length range of 25 mm–500 mm. The curve can be obtained by a high-order polynomial or exponential function fitting. However, a high-order polynomial increases the amount of DSP calculations, and a low-order polynomial affects the fitting accuracy. The experimental results show that the accuracy of the exponential fitting is not high for a wide focal length range.

#### 3.3.3. Levenberg–Marquardt Algorithm Identification Parameters

According to the relation between the potentiometer code values and focal length, the model is introduced in this project as follows:

$$F(X)=AX^{5}+BX^{4}+CX^{3}+DX^{2}+EX+G$$

where *A*, *B*, *C*, *D*, *E*, and *G* are the identification parameters, X is the zoom potentiometer code value, and F(X) is the focal length value.

The Levenberg–Marquardt formulas are as follows:

(8) $$CF_{V}{}^{\left(k+1\right)}=CF_{V}{}^{\left(k\right)}+h_{tm}$$

(9) $$h_{tm}=-(J^{T}J+\mu I{)}^{-1}J^{T}r$$

(10) $$r_{i}=Y_{i}-F_{i}(X_{i},A,B,C,D,E,G)$$

(11) $$e=\frac{1}{2}{\sum_{i=1}^{43}\left(r_{i}\right)}^{2}$$

(12) $$J=[\frac{\partial e}{\partial A},\frac{\partial e}{\partial B},\frac{\partial e}{\partial C},\frac{\partial e}{\partial D},\frac{\partial e}{\partial E},\frac{\partial e}{\partial G}]$$

where $Y_{i}$ is the actual measured focal length value, $X_{i}$ is the zoom potentiometer code value, and $CF_{V}={\left[A,B,C,D,E,G\right]}^{T}$.

The iterative steps are as follows:

Step 1: The initial coefficient value is $CF_{V}{}^{\left(0\right)}=[-1\times 10^{-11},1\times 10^{-8},-1\times 10^{-5},0.01,-1,100]$, the radius is μ=0.01, the parameter dimension is 6, the number of data is 43, and the maximum iteration is 30.

Step 2: The error of the result between the current coefficient model and the measured focal length result is calculated to be $r_{i}{}^{old}$.

Step 3: The Iteration Formula (8) is used to update the coefficients A, B, C, D, E, and G.

Step 4: The error of the result between the update coefficient model and the measured focal length is calculated to be $r_{i}{}^{new}$.

Step 5: If $r_{i}{}^{new}>r_{i}{}^{old}$, then μ=2×μ, and the model coefficient is updated.

Step 6: If the algorithm converges, the convergence condition is $\left|CF_{V}{}^{\left(k+1\right)}-CF_{V}{}^{\left(k\right)}\right|<10^{-5}$; if it does not converge, then we return to Step 2.

Step 7: The algorithm ends after 30 instances of operation.

We used the Levenberg–Marquardt iterative algorithm for fitting, and the results show that the chi-squared tolerance value of $10^{-9}$ was reached after 13 iterations. It has fast convergence and high fitting accuracy.

### 3.4. Autofocusing Control Algorithm

#### 3.4.1. Improving the Area Search Algorithm

**Figure 10:**
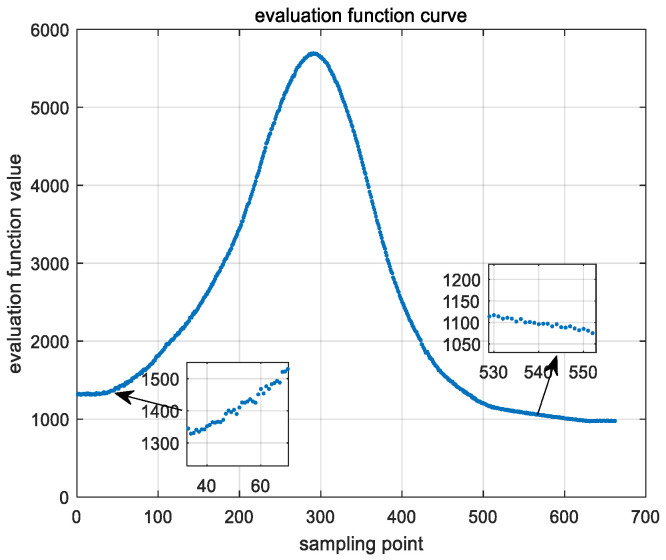
Evaluation function curve.

Based on the traditional mountain-climb method, the improved area search method is adopted to find the evaluating function extreme point. A modified backlash value is added to compensate for the gear gap. When the focusing motor drives the lens group motion, the gray value of the image obtained by the CCD camera changes as the scene changes, and the values of the evaluation function also change. The overall trend of the actual evaluation function is an approximate parabola, as shown in Figure 10. From the enlarged figure in Figure 10, we can see that the values significantly fluctuate on both sides of the evaluation function curve. Although the target is fuzzy in the actual tracking process, it is not on both sides of the evaluation function, so it is unnecessary to consider the extreme condition. From the enlarged figure in Figure 10, there is a small mutation in the middle of the curve, and mutation can be avoided through the improved area search method.

#### 3.4.2. Improving the Autofocusing Algorithm Implementation Process

**Figure 11:**
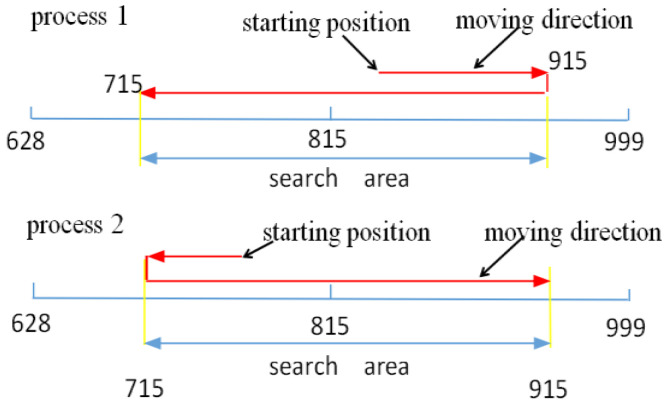
Autofocusing process diagram.

The focusing potentiometer whole code values range from 628 to 999. Therefore, the focusing process is divided into two parts in terms of the focusing lens group starting position. If the starting position was located between 815 and 999, we applied Process 1, which is displayed in Figure 11, to search the evaluation function’s extreme value; otherwise, we applied Process 2. However, in a practical project, we reduce the search range to avoid falling into a local extreme value at the two sides of the evaluation function and also to improve the search speed. The search process diagram is shown in Figure 11.

In Figure 11, if the focusing lens group is located on both sides, it will fall into the local extreme value. Therefore, we designed the search area according to the field experiment. Finally, the extreme value is found, and the program causes the focusing lens group to stop at the extreme position, that is, the clear part of the image. In actual work, the lens group cannot accurately stop at the extreme position due to the influence of the gear gap, but it stops near the extreme position. The backlash can be tested by experiment; then, the lens group can stop at the extreme position after increasing the fixed deviation in the position process. In the actual tracking target process, the target in the field of view after blurring and the focusing lens group is located at the near extreme point of the evaluation function and will not fall into the local extreme value. The target can be quickly recaptured through autofocusing.

**Figure 12:**
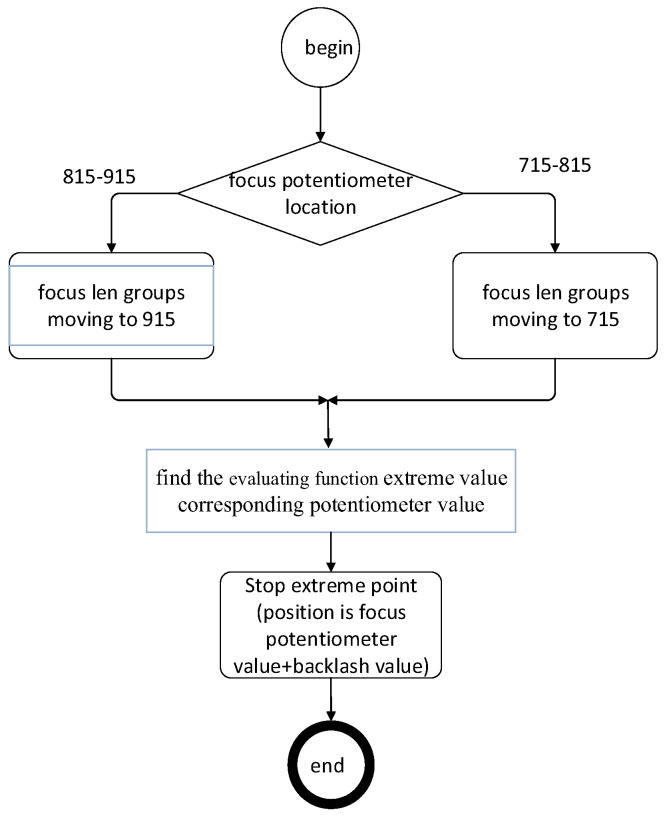
Auto focus algorithm flow chart.

The DSP AD module collected the code range of the focusing potentiometer from 628 to 999. To prevent the mutation of the evaluation function values in a small range and to search for the evaluation function value simultaneously, we use the area search method to improve the search speed. The flow chart of the autofocusing program is shown in Figure 12.

## 4. Experimental Results

### 4.1. Improved RBF ANN Fuzzy Control Experiment

**Figure 13:**
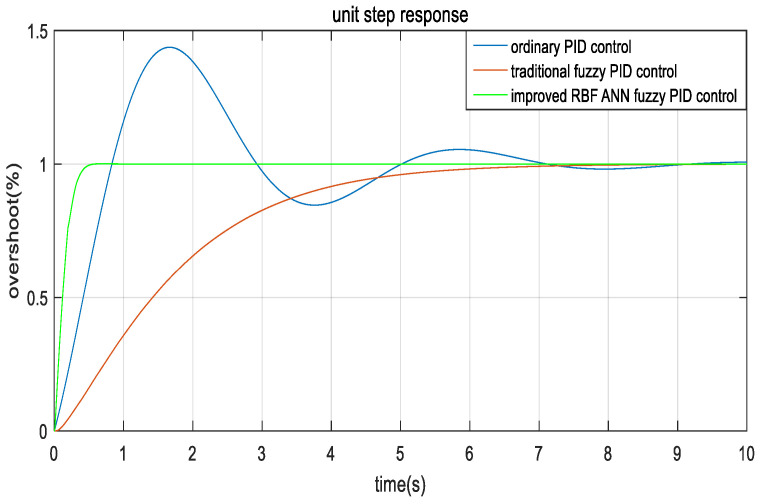
Unit step response comparison diagram.

In the traditional fuzzy PID control system, the PID parameter is set to $k_{p}=6$, $k_{i}=15$, and $k_{d}=5$. The unit position step response simulation is compared with the proposed RBF ANN fuzzy PID control system in MATLAB. A step response comparison diagram is shown in Figure 13.

Figure 13 shows that the ordinary PID control algorithm performance index ITAE is 2.545, and the settling time is 9 s. The traditional fuzzy PID control algorithm ITAE is 2.704, and the settling time is 7 s. The ITAE of the improved RBF ANN fuzzy PID control algorithm is 0.01603, and the settling time is 0.6 s. The traditional fuzzy PID control algorithm and RBF ANN fuzzy PID control algorithm have almost no overshoot.

According to the simulation analysis of the step response, we proposed that the control algorithm performance index is improved by more than two orders of magnitude compared with the traditional fuzzy PID control algorithm, and the settling time is 6.4 s faster than the traditional fuzzy PID control.

### 4.2. Continuous Zoom Experiment

**Figure 14:**
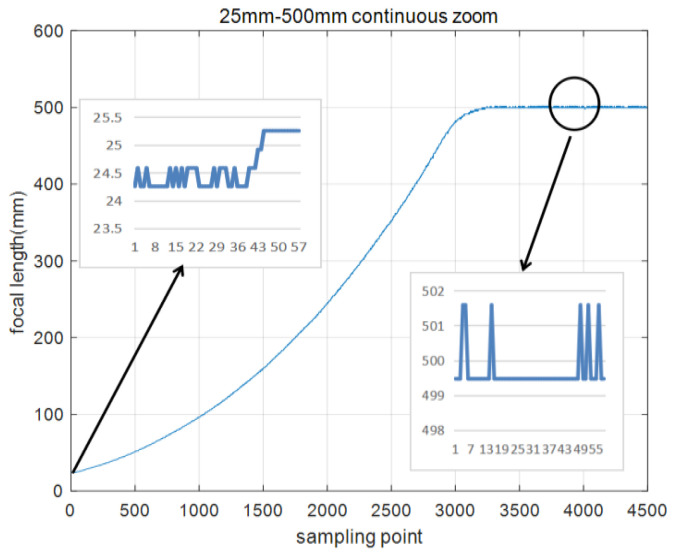
Zoom process from 25 mm to 500 mm.

**Figure 15:**
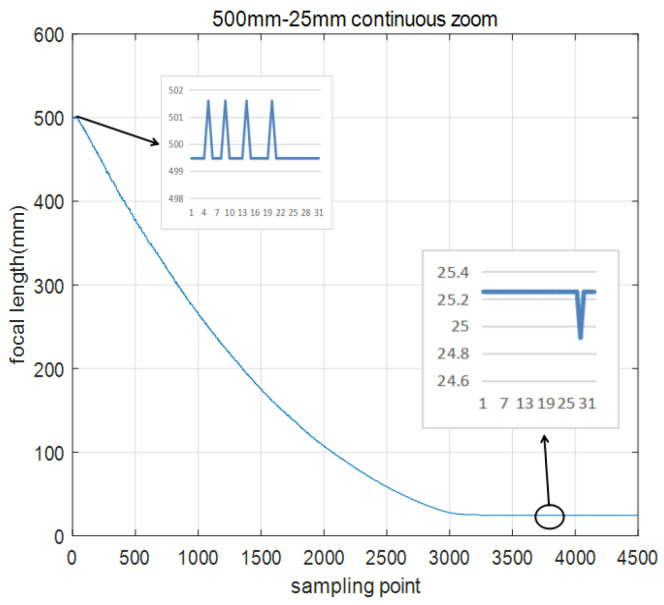
Zoom process from 500 mm to 25 mm.

In the actual continuous zoom process, the DSP in-chip RAM records the focal length values for 4500 frames, and the sampling frequency is 800 Hz. The zoom process from a large field of view to a small field of view starts at 25 mm and ends at 500 mm, as shown in Figure 14. The opposite process is shown in Figure 15.

Figure 14 and Figure 15 show that the RBF ANN fuzzy PID control algorithm has almost no overshoot, and the zoom process from 25 mm to 500 mm requires 4.04 s. The opposite process is 3.92 s. Due to the existence of the gear gap, the zoom process time is different. From the enlarged figures in Figure 14 and Figure 15, the steady-state errors are very small. There are three main points. 1. In the zoom position closed-loop control process, the maximum steady-state errors between a given focal length and feedback focal length are 3 code values. 2. There is a focal length fitting error. 3. Due to the gear gap, the regulator integral action constantly adjusts the zoom motor, resulting in errors.

### 4.3. The Focal Length Fitting Experiment

**Figure 16:**
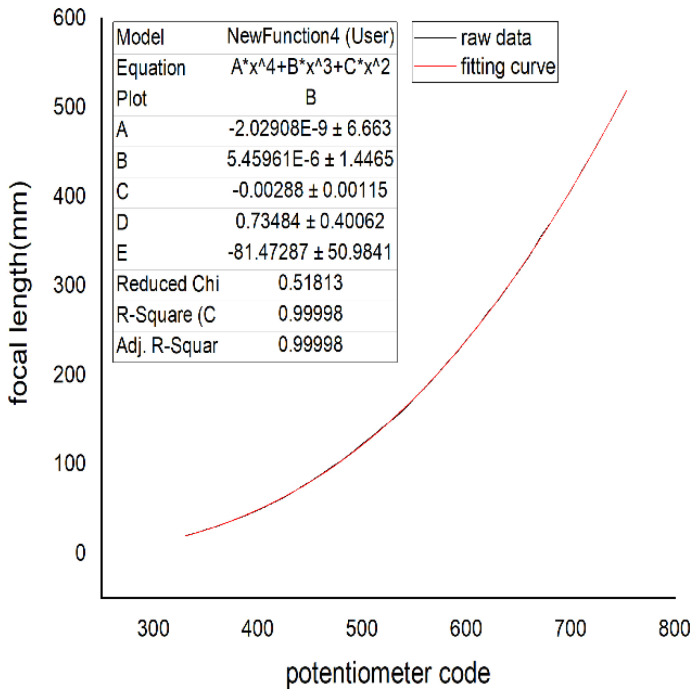
Quartic polynomial fitting.

**Figure 17:**
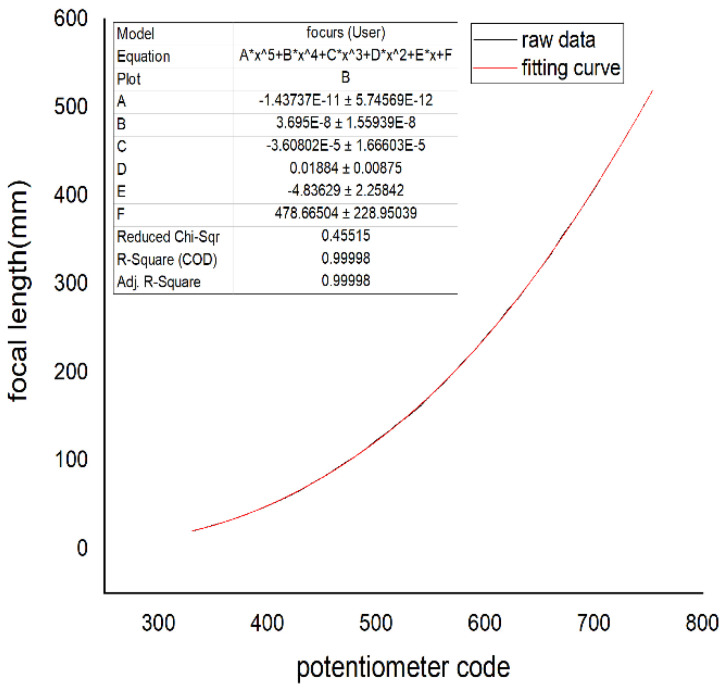
Quintic polynomial fitting.

**Figure 18:**
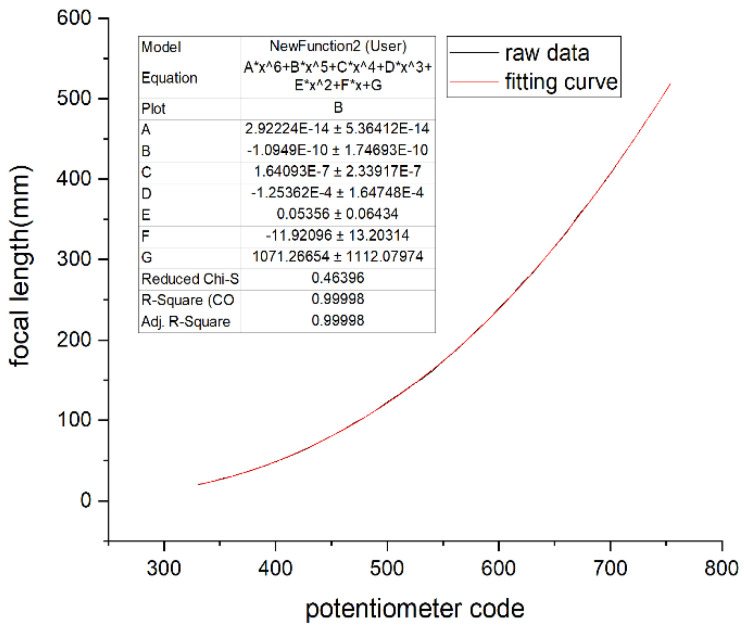
Sixth degree polynomial fitting.

**Figure 19:**
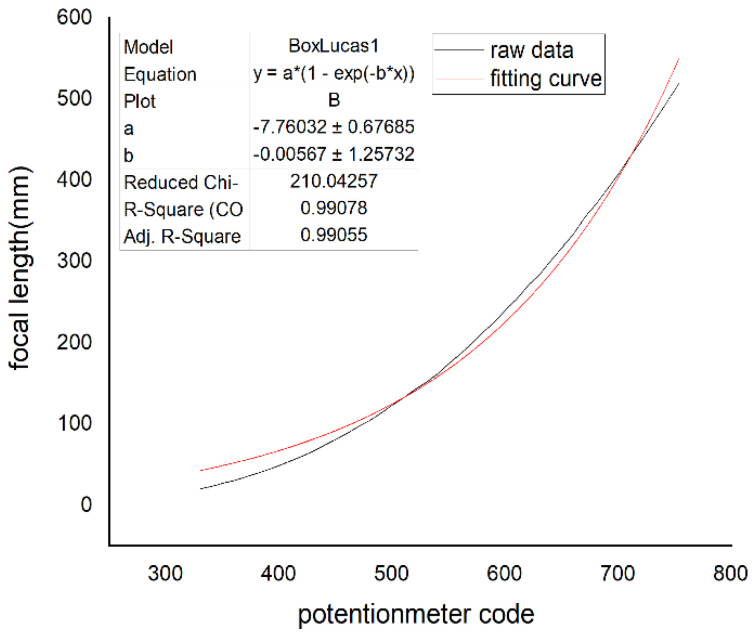
Exponential fitting.

The four curves of the focal length and potentiometer code values fitted by the Levenberg–Marquardt algorithm are shown in Figure 16, Figure 17, Figure 18, and Figure 19, respectively.

In Figure 16, Figure 17, Figure 18 and Figure 19, the horizontal ordinate is the zoom potentiometer code value, and the vertical coordinate is the focal length value. From the figures, we can see that the quartic, quintic, and sixth-degree polynomial and exponential fitting residual sums of squares are 0.51813, 0.45515, 0.46396, and 210.04257, respectively. The fitting results show that the fifth-degree polynomial residual sum of squares is minimal and that the fitting precision is high. The sixth-degree polynomial residual sum of squares is almost the same as that of the fifth-degree polynomial. If the sixth-degree polynomial fitting function is adopted, it will increase the DSP computation. Due to the wide range of focal length variation, the traditional exponential fitting function accuracy is low and cannot be applied to the wide range of focal length fitting. The quintic polynomial focal length fitting can completely cover the range from 25 mm to 500 mm. It meets engineering requirements. To verify the output focal length accuracy of the fitting curve, the eight groups of focal length data were tested, and the average error was taken 10 times in each group. Given 25, 50, 80,100, 200, 300, 400, and 500 focal length instructions in the position closed-loop control zoom lens group, we used a similar triangle method to test the camera focal length value. Finally, we compared the focal-length accuracy with two methods. The results are shown in Table 2.

### 4.4. Autofocusing Experiment

**Figure 20:**
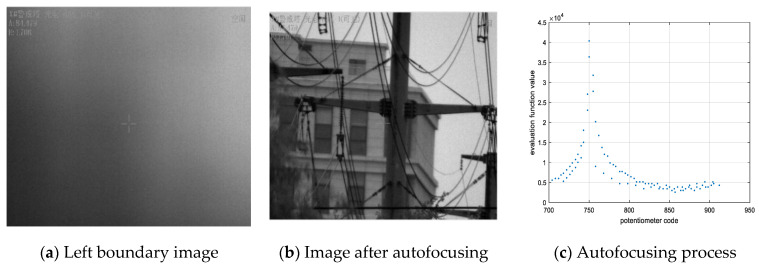
Left boundary autofocusing process.

**Figure 21:**
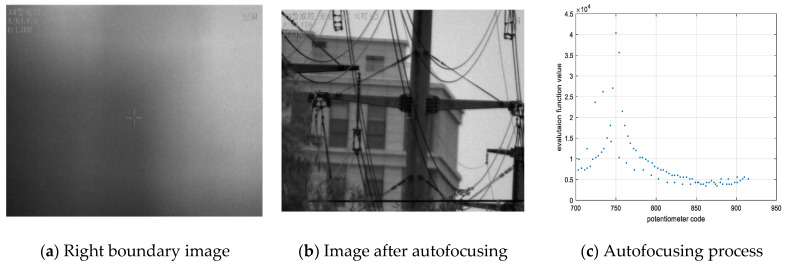
Right boundary autofocusing process.

We found that if the range of the focusing potentiometer can guarantee a clear image obtained by autofocusing at a focal length of 500 mm, then a clear image can be obtained from focal lengths of 25 mm to 500 mm. Therefore, a visible-light camera autofocusing experiment is conducted on a 4.2 km target in this project, and the boundary value of the autofocusing lens group is found when the focal length is 500 mm. The range of the focusing lens group potentiometer is 628 to 999, but it was found that the extreme value of the evaluation function was always located between 715 and 915 for different scenes and at different times. Therefore, to improve the search speed, the search range is shortened during the autofocusing process. At the same time, the autofocusing algorithm can avoid local extrema because of the focusing lens group at both ends of the evaluation function curve. To test the maximum search time, the autofocus lens polarity was manually adjusted to the left and right limit boundaries. The blurred image when the focus lens group is on the left side of the limit is shown in Figure 20a. The autofocusing image is shown in Figure 20b, and the autofocusing process is shown in Figure 20c. The right boundary autofocusing process is shown in Figure 21.

In Figure 20, we can see that the focusing potentiometer left boundary value is 715, and autofocusing occurs. The focusing lens groups move to the right limit boundary and then return to the left limit boundary. Finally, the focusing mirror group is positioned to the extreme value of the evaluation function. The extreme value of the evaluation function is 40,373 after autofocusing, and the corresponding potentiometer value is 750. In the autofocusing process, the amount of data collected by the main control computer is 92, and the collection frequency is 50 Hz; thus, the autofocusing time is 1.84 s. In Figure 21, we can see that the focusing potentiometer right boundary value is 915, and autofocusing occurs. The focusing lens groups move to the left limit boundary and then return to the right limit boundary. Finally, the focusing mirror group is positioned to the extreme value of the evaluation function. The extreme value of the evaluation function is 40,298 after autofocusing, and the corresponding potentiometer value is 750. In the process of autofocusing, the amount of data collected by the main control computer is 80, and the collection frequency is 50 Hz; thus, the autofocusing time is 1.6 s. Because the mechanical structure of the camera control system is fixed, every autofocusing time is the same, basically under the same position in position and velocity double closed-loop system. From the experimental results, we can see that the left and right boundaries of the focusing mirror group can return to the extreme value position of the evaluation function after the correction backlash. Therefore, the focusing lens group can autofocus and obtain clear images, in which the range of the potentiometer values is from 715 to 915. In the experiment, we set the left and right limit boundary positions to start autofocusing, but the starting point is actually located somewhere between 715 and 915, and the starting direction is set to move to the nearest boundary in the program. In other words, when the autofocusing starting position is located between 715 and 815, the lens groups move toward the potentiometer value of 715 and then back to 915. Moreover, the mirror group locates the extreme value. If the autofocusing starting position is located between 815 and 915, the lens groups move toward the potentiometer value of 915 and then back to 715. Additionally, the mirror group locates the extreme value. Therefore, the autofocusing time must be less than 1.84 s. The traditional autofocusing search full range is from 628 to 999; after improvement, the search range is from 715 to 915. Therefore, the search area is almost shortened to half; thus, the autofocusing time is decreased by more than half that of the traditional method. It is worth noting that the mirror group is out of the range of 715 to 915, and autofocusing will not be achieved. However, in the process of tracking the target, the mirror group will not be beyond this range.

## 5. Conclusions

The improved RBF ANN fuzzy control algorithm is applied to position and velocity double closed-loop camera control systems, and it has almost no overshoot. The ITAE performance index is improved by more than two orders of magnitude compared with the traditional fuzzy PID control algorithm, and the settling time is 6.4 s faster than the traditional fuzzy PID control, which meets the requirements of camera system positioning. The Levenberg–Marquardt iterative algorithm has a fast convergence speed, and the fitting precision is high. Compared with traditional exponential fitting, the proposed method is improved by more than three orders of magnitude. It meets engineering requirements with a wide range of focal lengths from 25 mm to 500 mm. Under the complex background and small field of view, the autofocusing time is less than 2 s, and it is improved by more than double that of the traditional method. Therefore, the improved search method can quickly achieve autofocusing to obtain a clear image and compensate for backlash. The proposed method can also be applied to infrared camera control systems.

## Figures and Tables

**Table 1:** Camera parameters.

Frame frequency	50 Hz
Pixels	1280×1024
Pixel Size	4 μm
Focus Length	25−500 mm
Aperture	80 mm

**Table 2:** Focal length precision.

Serial Number	Given Focal Length (mm)	Traditional Fuzzy PID Control Average Error (mm)	Improved RBF ANN Fuzzy PID Control Average Error (mm)
1	25	0.04	0.02
2	50	0.09	0.05
3	80	0.08	0.04
4	100	0.07	0.03
5	200	0.03	0.01
6	300	0.05	0.02
7	400	0.04	0.02
8	500	0.03	0.01

## Data Availability

The data used to support the findings of this study are available from the corresponding author upon request.
